# Uncovering Structural Plasticity of *Enterovirus A* through Deep Insertional and Deletional Scanning

**DOI:** 10.21203/rs.3.rs-3835307/v1

**Published:** 2024-01-24

**Authors:** William Bakhache, Walker Orr, Lauren McCormick, Patrick T. Dolan

**Affiliations:** 1. Quantitative Virology and Evolution Unit, Laboratory of Viral Diseases, NIH-NIAID Division of Intramural Research, Bethesda, MD, USA; 2. Department of Biology, University of Oxford, Oxford, UK

## Abstract

Insertions and deletions (InDels) are essential sources of novelty in protein evolution. In RNA viruses, InDels cause dramatic phenotypic changes contributing to the emergence of viruses with altered immune profiles and host engagement. This work aimed to expand our current understanding of viral evolution and explore the mutational tolerance of RNA viruses to InDels, focusing on Enterovirus A71 (EV-A71) as a prototype for Enterovirus A species (EV-A). Using newly described deep InDel scanning approaches, we engineered approximately 45,000 insertions and 6,000 deletions at every site across the viral proteome, quantifying their effects on viral fitness. As a general trend, most InDels were lethal to the virus. However, our screen reproducibly identified a set of InDel-tolerant regions, demonstrating our ability to comprehensively map tolerance to these mutations. Tolerant sites highlighted structurally flexible and mutationally plastic regions of viral proteins that avoid core structural and functional elements. Phylogenetic analysis on EV-A species infecting diverse mammalian hosts revealed that the experimentally-identified hotspots overlapped with sites of InDels across the EV-A species, suggesting structural plasticity at these sites is an important function for InDels in EV speciation. Our work reveals the fitness effects of InDels across EV-A71, identifying regions of evolutionary capacity that require further monitoring, which could guide the development of Enterovirus vaccines.

## Introduction

In RNA viruses, non-synonymous mutations are the primary mechanism of adaptation to selective pressures on short evolutionary timescales ^1,2^. On longer timescales, rarer insertion, deletion, and recombination events occur, which can confer much greater phenotypic effects, potentially altering protein function or structure in more profound ways ^3^. Both of these mutational forces, one occurring quickly with usually subtle effects, and the other, driving more significant changes in virus biology, combine to set the tempo of the host-virus arms race ^4^. Therefore, mapping the evolutionary potential of InDels in viral proteomes is of fundamental importance to our understanding of viral evolution and emergence.

Viruses in the order *Picornavirales* abound in our natural world, infecting a wide range of uni- and multicellular organisms. Picorna-like viruses are proposed to be ancestral to all modern viruses, imparting the core structural feature of icosahedral capsid proteins, the ‘jelly roll’ fold, and numerous non-structural modules involved in viral replication ^5,6^. One family of *Picornavirales*, the Enteroviruses include over 40 genera of human pathogens that exhibit a wide range of tissue tropisms, modes of pathogenesis, and immune profiles; and that pose significant public health challenges ^7,8^.

While the core features of the viral proteome are conserved across this important class of pathogens, their variability in tropism and pathogenesis present important questions about how viral proteins evolve and diversify. The importance of InDels in *Picornavirales* evolution is exemplified by the emergence of Rhinovirus C. InDels in the capsid protein dramatically changed its surface topography ^9,10^, shifting it to use a new receptor, CDHR3, an event which altered its immunological profile and shaped human evolution ^11^. Despite detecting historical InDel events in Enteroviruses, a more comprehensive study of the fitness effects of these mutations on any viral proteome has not been performed.

Deep Mutational Scanning (DMS) techniques have been transformative in understanding the effects of mutation, allowing measurement of the fitness effects of all possible non-synonymous mutations across cellular proteins ^12,13^. In viral proteins, DMS studies have identified the biological and biophysical constraints shaping mutational tolerance ^14–18^ and the mutational pathways available to escape immune pressures ^16^. However, DMS is limited to single residue substitutions and does not identify regions tolerant to sequence insertion or deletion. Owing to technical limitations in generating InDel libraries, previous studies examining the effects of InDels on viral fitness have either relied on high-fidelity population sequencing ^2^ or random transposon insertion mutagenesis ^19,20^. Deep Insertional and Deletional Scanning approaches have been recently developed to overcome these limitations, Saturated Programmable Insertion Engineering (SPINE) and Deep Insertion, Deletion, and Missense Programmable Library Engineering (DIMPLE) ^21,22^, which have been used to explore insertional and deletional tolerance in cellular proteins.

One species of Enterovirus of particular public health concern is the Enterovirus A species (EV-A), which circulate worldwide causing large outbreaks of hand, foot, and mouth disease among young children ^7^, however, some genotypes, such as EV-A71, can also cause severe neurological complications such as meningitis and brainstem encephalitis that can be fatal ^23,24^. EV-A71 has been therefore designated as one prototype pathogen for understanding the biology, immunology, and evolution of Enteroviruses with pandemic potential ^8^.

In this work, we aimed to comprehensively address the role of InDels in the evolution of EV-A species. We implemented the newly developed deep InDel scanning approaches to characterize the fitness effects of InDels in the EV-A71 proteome. We used the programmable nature of this approach to modify insertion sequence and deletion length, allowing characterization of the impact of these changes on InDel fitness effects. We mapped the relative fitness effects of InDels onto the structures of the capsid and non-structural proteins to provide a physical interpretation of regions with differential tolerance. Finally, we applied a phylogenetics-based approach to uncover the historical tolerance of EV-A species to InDels and identify a region in the N-terminus of the VP1 capsid protein, where these mutations occur frequently. This work sheds light on the role of InDels in the long-term evolution of Enteroviruses. The patterns of constraint uncovered in our study will help to understand the forces that drive the emergence of Enteroviruses, and could guide the development of Enterovirus vaccines with broad immunogenic properties ^25^.

## Results

### Deep Insertion and Deletion Scanning across the EV-A71 proteome

**Figure 1.**
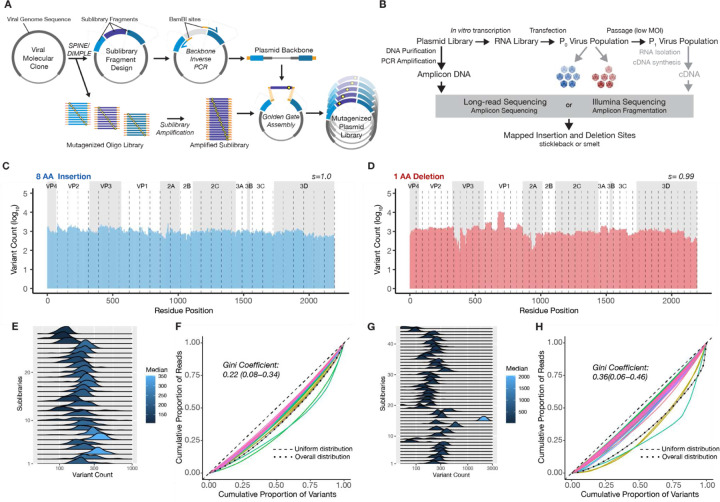
Deep InDel scanning of the EV-A71 proteome (a) Molecular and computational pipeline for the generation of deep Indel scanning libraries. (b) The experimental virology pipeline for the rescue of viruses from molecular clones and the subsequent sequencing at different steps of the process. Bar plot (bin=5) showing the variant count across the viral proteome for insertions (c) and deletions (d). The distribution of variants is shown through ridge kernel line plots and Lorenz curves for insertions (e,f) and deletions (g,h). s (saturation) is the proportion of detected variants to designed variants.

To understand the global tolerance of the EV-A71 proteome to insertions and deletions, we performed a deep InDel scanning experiment across the full EV-A71 coding sequence, which is expressed as a single polyprotein of 2193 amino acid (AA) residues. We used previously reported pipelines, SPINE and DIMPLE ^21,22^, to computationally divide the polyprotein coding sequence of the EV-A71 Tainan/4643/98 molecular clone (Genbank accession: AF304458.1) into ‘sublibrary fragments’ (Figure 1 A). These pipelines then generate two outputs: i.) oligonucleotide primers to amplify the unmutagenized backbone sequence flanking each sublibrary fragment, and ii.) oligonucleotide sequences corresponding to each sublibrary fragment, encoded with the defined library of variants. Primers and oligonucleotide pools are flanked with BsmBI restriction enzyme sites to facilitate the assembly of the mutagenized oligonucleotide libraries with the unmutagenized plasmid backbone by Golden Gate assembly ^26^.

Two separate strategies were used to generate InDel libraries. Deletional libraries, encoding deletions of 1, 2, and 3 codons across the entire polyprotein open reading frame were directly encoded in our oligonucleotide pool as 45 sublibrary fragment assemblies, each assembled into the corresponding backbone. Insertional libraries were generated by directly encoding an in-frame peptide sequence, known as an ‘insertional handle’, at each codon position in the oligonucleotide pool as 28 sublibrary fragment assemblies. The handle contains two outward-facing BsaI restriction enzyme sites that facilitate the subsequent cloning of any sequence of interest into each handle site (Supp. Figure 3 A). This enables the rapid generation of insertional libraries containing a diverse range of inserts once the initial handle plasmid library is constructed.

Mutagenized libraries and corresponding rescued and passaged viruses were sequenced either by long-read nanopore sequencing (for large insertions) or short-read sequencing (for small InDels) (Figure 1 B). Our sequence analysis (Supp. Figure 1 A, B) reveals the libraries are nearly complete, with 99.70% of the insertion and deletion variants detected at least once (Figure 1 C, D and Supp. Figure 2 A, B). The distribution of variant counts within different sublibraries was visualized using ridgeline kernel density plots and Lorenz curves ^27^, revealing a balanced distribution (Figure 1 E, G and Supp. Figure 2 C, E). Some variability in loading between sublibraries is shown as shifts in the overall distribution, with medians of sublibraries varying only across an approximately three-fold range for insertions, and a six-fold range for deletions. We quantified the balance of reads between variants using the Gini coefficient, a measure of inequality scaled from 0 (uniform) to 1 (a single variant in all reads) ^28^, obtaining a value of 0.22 for insertion libraries and 0.34–0.36 for deletion libraries (Figure 1 F, H and Supp. Figure 2 D, F). These Gini coefficients were consistent with libraries generated previously by this method ^22^.

To explore the influence of insertional sequence composition on insertional tolerance, we engineered a variable peptide sequence into each codon position via the insertional handle. The inserted sequence consisted of each of the 20 amino acids flanked by flexible linkers. This insertion library was nearly complete, detecting 96–99% of variants, with median variant counts of each peptide sequence spreading across a two-fold range (Supp. Figure 3 B, C).

Due to improvements in efficiency and library quality provided by the synthetic approach to library generation, SPINE and DIMPLE consistently produced saturated and balanced InDel libraries on our relatively large target open reading frame. The modularity and efficiency of this pipeline allowed the engineering of InDel libraries in 1–2 months from design to virus rescue. With the added efficiency of the insertional handle strategy to generate new insertional libraries, we can produce a viral library with a new insertion sequence at each handle position in a matter of days.

### InDel fitness effects across the EV-A71 proteome

**Figure 2.**
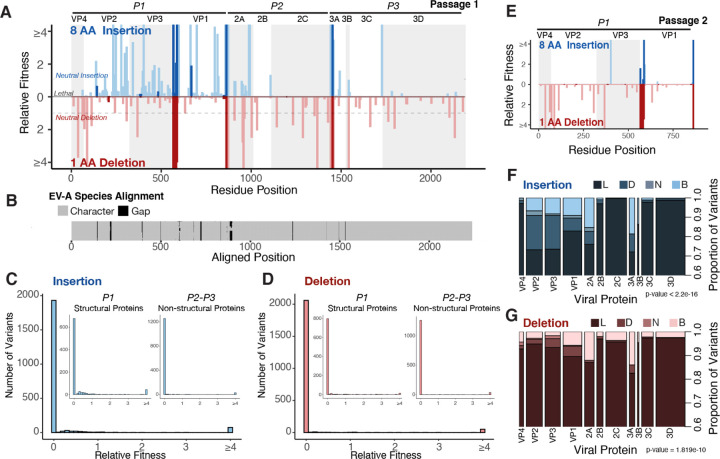
InDel fitness effects across the EV-A71 proteome (a) Bar plot (bin=5) showing the relative fitness of insertion and deletion variants across the viral proteome. RD cells were infected with insertion and deletion passage 0 virus libraries at an MOI≤0.1 to produce passage 1. Dashed lines represent neutral InDels where 0.9≥ω≤1.1 and lethal ω=0. Darker shades of blue and red represent regions overlapping with natural InDels. (b) Graphical representation of gaps in a multiple sequence alignment of all EV-A species. Histograms visualizing the distribution of mutational fitness effects for insertions (c) and deletions (d). Inset histograms show the distribution for structural and non-structural proteins. (e) Bar plot (bin=5) showing the relative fitness of insertion and deletions variants across the capsid proteins at passage 2. Area plot showing the proportion of variants within a fitness class for insertions (f) and deletions (g) for the different viral proteins. The width of the column represents the total number of variants for a given protein. Chi-squared statistics for (f) degrees of freedom (df)=30, chi-squared=345.4 and for (g) df=30, chi-squared=106.32.

After validating the quality of our InDel libraries, we generated passage 0 virus populations by transfecting *in vitro* transcribed viral RNA into RD cells. Passage 0 virus titers ranged from 10^3^–10^4^ TCID_50_/mL for insertion libraries and 10^5^–10^6^ TCID_50_/mL for deletion libraries (Supp. Figure 4 A, B). Then, we passaged the virus at a low multiplicity of infection (MOI≤0.1) and sequenced the corresponding virus population at 9 hrs post-infection. We calculated the relative fitness values (*ω*) of InDels by normalizing the frequency of each engineered variant in the virus population to its frequency in the input plasmid population (Figure 2 A). We found insertion and deletion mutations were tolerated at shared hotspot regions. Notably, these hotspots for InDels overlapped substantially with InDels occurring during the diversification of the EV-A species (Figure 2 A: darker shades, and Fig 2 B). In the P1 capsid proteins, the N-terminus of VP1 exhibited the highest tolerance to InDels, where residue 585 and 579 showed the highest relative fitness for insertions and deletions, respectively. In the non-structural proteins, the N-terminus of 2A was a hotspot region for InDels, where residue 863 had the highest relative fitness for insertions and position 864 for deletions. To validate our pipeline, we generated five viral molecular clones with an insertional handle at different positions in the capsid proteins. The calculated relative fitness values in the insertional screen correlated with a miniature screen and passage 0 titers for these variants, showing the robustness of the deep Indel fitness values (Supp. Figure 4 C–F).

Visualizing the distribution of mutational fitness effects (DMFE) highlighted that most InDels are lethal to the virus, with only a small number of variants tolerated (Figure 2 C, D). This is distinct from the bimodal DMFE for single nucleotide variants (SNVs) previously described from sequencing of viral populations ^29–31^. Despite the lower rate at which InDels occur, about 5–10 fold lower than SNVs, this lethal bias places a significant deleterious load on the viral population, consistent with our previous studies describing the DMFE of naturally occurring InDels in experimental RNA virus populations ^2^.

We next classified variants into fitness classes (Beneficial *ω*>1.1, Neutral 0.9≥*ω*≤1.1, Deleterious *ω*<0.9, and Lethal *ω*=0) and compared the proportion of variants in each fitness class for each viral protein. This analysis highlighted VP1, 2A(pro), and 3A as the most robust to InDels, having the highest number of beneficial and neutral variants (Figure 2 F, G). The two largest replication proteins, 2C and 3D, were the least tolerant to insertions, having a smaller proportion of beneficial and neutral variants. Overall, our pipeline yields quantitative data about the fitness effects of InDels across the EV-A71 proteome, identifying key tolerated regions present mainly in VP1, 2A(pro), and 3A.

Complementation of capsid mutations can occur between trans-encapsidation of the mutant genome in an alternative capsid protein. Therefore, we generated passage 2 of our InDel capsid libraries at low MOI. The hotspot regions detected in passage 1 were also in passage 2 (Figure 2 E). Interestingly, mutations in the passage 1 libraries were not uniformly rescued through complementation, suggesting the large extent to which InDels likely act as dominant negatives in the packaging or entry process.

### Impact of altering insertion sequence and deletion length on InDel fitness effects

**Figure 3.**
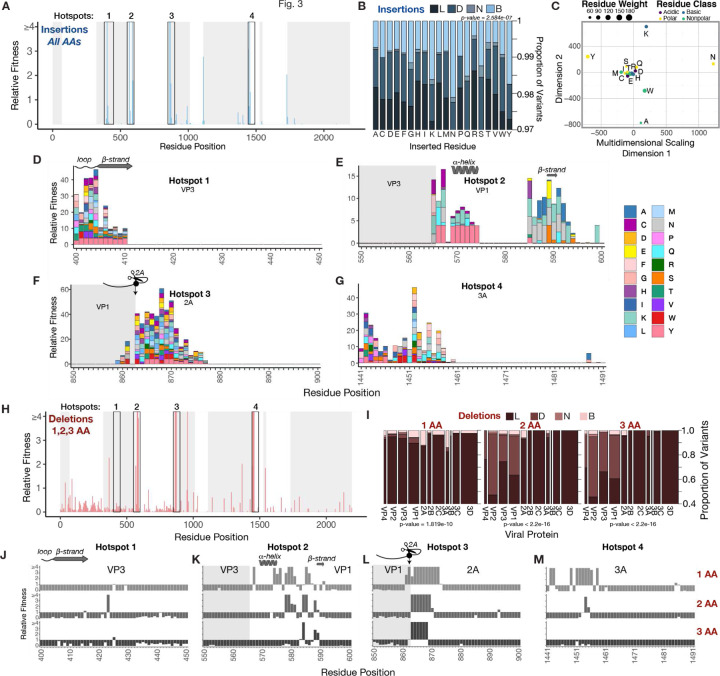
Impact of altering insertion sequence and deletion length on InDel fitness effects (a) Mean relative fitness for all different amino acid insertions is shown in the barplot (bin= 5). (b) Area plot showing the proportion of variants within a fitness class for all the different inserted residues. Chi-squared=127.52 and df=57. (c) Projection of a multidimensional dataset to two dimensions through multidimensional scaling. Residue weight is represented by the size of the circle, and residue class by the color of the circle. (d-g) Bar plots (bin=1) of zoomed in regions from 1–4 showing the relative fitness of the different inserted residues at these positions. (h) Mean relative fitness for all three types of deletions is shown in the barplot (bin=5). (i) Area plot was used to show the proportion of variants in a specific fitness class for deletions of different sizes. Chi-squared statistics for 1AA (chi-squared =106.32, df=30), 2 AA ( chi-squared=675.7, df=33), and 3 AA (chi-squared =692.94, df=30). (j-m) Zoomed in bar plots (bin=1) on the regions 1–4.

Insertional tolerance can also be influenced by the composition of the inserted sequence. Therefore, we characterized the fitness effects of a library variable 5-residue peptides encoding each of the 20 amino acids flanked by a flexible linker. The mean fitness effects of all variants were plotted, revealing hotspot regions that aligned with the insertional handle results (Figure 3 A). Analysis of the distribution of variants across fitness classes showed differential tolerance for different amino acids. Arginine exhibited the lowest proportion of beneficial variants, while tyrosine, asparagine, and lysine demonstrated the highest tolerance (Figure 3 B).

To compare tolerance patterns between residues, we used multidimensional scaling to reduce the dimensionality of the positional tolerance data for each variant peptide (Figure 3 C). This revealed several distinct profiles of tolerance for specific residues, separating lysine, tyrosine, asparagine by their eccentric behavior in the analysis. Alanine and tryptophan clustered together consistent with their shared non-polar character. Insertions carrying lysine, tyrosine, or asparagine residues in the variable position of the inserted peptide were more context specific, possibly reflecting the chemical characteristics as well as post-translational modifications of the peptides after insertion, which may enhance or inhibit viral functions.

Sequence context-specific factors also accounted for differences in insertional tolerance for a specific variable peptide. We visually represented the fitness effects for the different insertion sequences in four hotspot regions (Figure 3 D–G). This showed that the N-terminus of VP1 is sequence-specific, tolerating a select few amino acids, with lysine, asparagine, and tyrosine being the most represented in this region (Figure 3 E). In contrast, a loop in VP3 and the N-termini of 2A and 3A exhibited broad tolerance to most amino acids (Figure 3 D, F, G).

Next, we evaluated the effects of deletion size on virus fitness, plotting the mean fitness effects of deletions across the EV-A71 proteome (Figure 3 H). The distribution of fitness effects for different deletion sizes showed that larger deletions yielded fewer beneficial variants (Figure 3 I). VP1 and 2A were the primary viral proteins exhibiting beneficial mutations for 2 and 3 AA deletion sizes. Plotting relative fitness values of different deletion lengths at the hotspot regions identified the N-terminus of VP1 and 2A as tolerant for all three deletion sizes (Figure 3 K, L). However, even at these sites, increased deletion lengths generally decreased fitness values. 3A showed higher sensitivity to deletion length with a noticeable drop in fitness values as deletion size increased (Figure 3 M).

In summary, a comprehensive analysis of all possible amino acid insertions highlighted the influence of amino acid properties on insertional tolerance, categorizing the detected hotspots as having either narrow or broad sequence specificity. Our ability to introduce various deletion lengths into our deep InDel scanning libraries underscored the variable tolerance of viral proteins to different deletion sizes, designating VP1 and 2A N-termini as highly tolerant for deletions of various lengths.

### Structural interpretation of InDel fitness values

**Figure 4.**
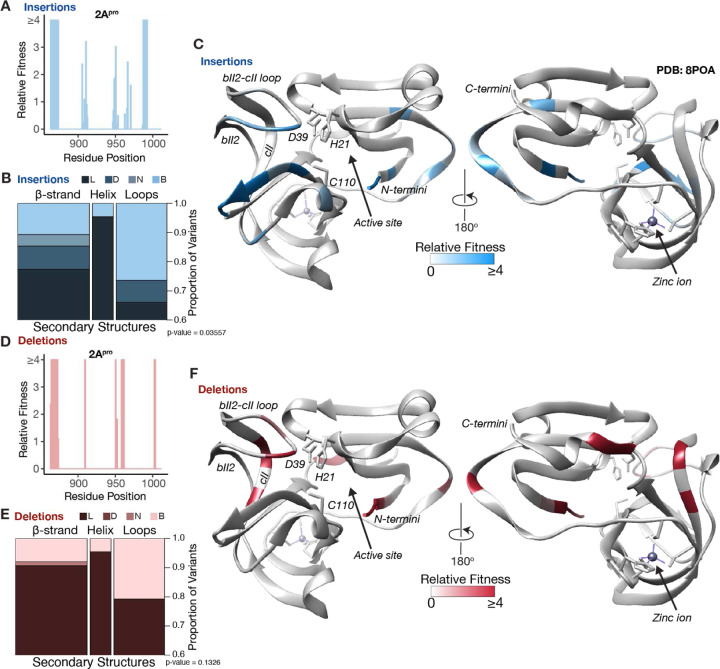
Structural interpretation of InDel fitness effects for 2A(pro) Bar plot (bin=1) for the relative fitness of insertions (a) and deletions (d) across 2A(pro). Area plot for the proportion of variants in a fitness class for the different secondary structures adopted by 2A(pro) for insertions (b) and deletions (e). Chi-squared statistics for (b) chi-squared=13.513, df=6 and (e) chi-squared =7.0625, df=4. Relative fitness values are displayed on the structure of 2A(pro) for insertions (c) and deletions (f). The PDB structure used for EV-A71 2A(pro) was PDB: 8POA.

To put our results in the structural context of EV-A71 proteins, we mapped the relative fitness values of InDels on resolved structures of the viral capsid and non-structural proteins. Initially, we mapped the fitness values onto the structure of the 2A(pro) non-structural protein, which exhibited the highest tolerance to InDels in our dataset (Figure 4 A, D). Functionally, 2A(pro) is the viral protease that cleaves the junction between the capsid and non-structural proteins. It also cleaves host proteins, contributing to the dampening of immune responses and the shutdown of host cap-dependent translation ^32^. 2A(pro) features an active site with a catalytic triad (H21, D39, and C110) and a zinc-finger binding domain. We classified our variants according to the secondary structures adopted by 2A(pro) ^33^. We observed that loops had the highest proportion of beneficial variants, defining them as optimal InDel sites (Figure 4 B, E). InDels in regions proximal to the active site and the zinc-finger binding domain were lethal to the virus (Figure 4 C, F), affirming the importance of these sites for 2A(pro) enzymatic activity. Beyond these critical sites, our analysis revealed regions tolerating InDels with the N-terminus and the bII2-cII loop emerging as prominent sites.

**Figure 5.**
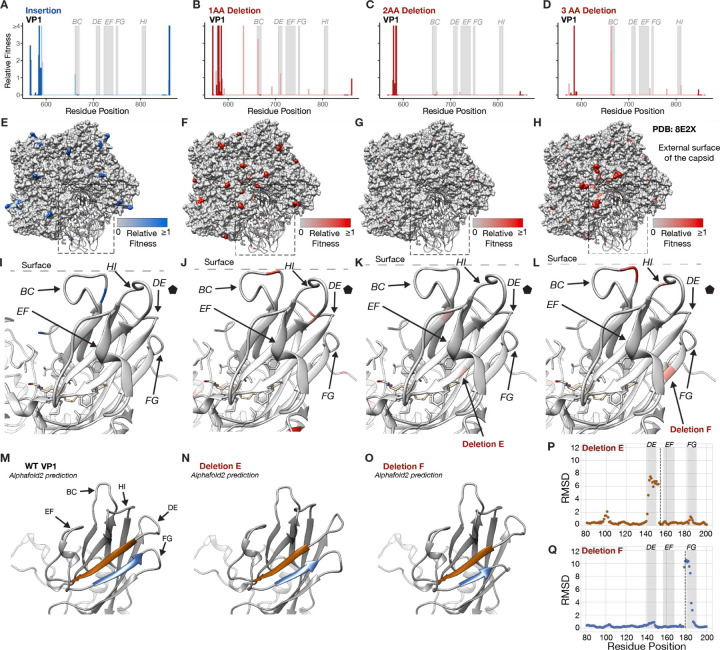
Structural interpretation of InDel fitness effects for VP1 Bar plots (bin=1) for insertions (a), 1 AA deletion (b), 2 AA (c), and 3 AA deletion (d) across the VP1 protein. Relative fitness values were mapped on the pentamer for insertions (e), 1 AA deletion (f), 2 AA deletion (g), and 3 AA deletion (h). (i-l) Zoom in on the previous panels focusing on the external loops of VP1. (m) AlphaFold2 prediction of VP1. AlphaFold2 prediction of VP1 with deletion E (n) and deletion F (o). RMSD comparison between the VP1 AlphaFold2 structure and VP1 with deletion E (p) and F (q). The PDB structure used for the EV-A71 virion was PDB: 8E2X.

The EV-A71 capsid comprises four capsid proteins (VP1 through VP4) initially assembling into a protomer. This protomer further organizes to form a pentamer and, ultimately, twelve pentamers come together to form the virion ^34^. In our structural analysis of the capsid proteins, we focused on VP1 since it had the highest tolerance to InDels after passage 2 (Figure 2 E). Tolerated InDels were predominantly located at the N-terminus of VP1 situated at the pentamer’s internal surface (Figure 5 A–D). However, the surface-exposed region of VP1 also exhibited tolerance to InDels, particularly at the BC loop, DE loop, and C-terminus. Variability of InDel tolerance at surface-exposed regions was observed when comparing insertions and deletions of different lengths (Figure 5 E–H). Remarkably, the BC loop displayed distinct tolerance, accommodating mainly deletions of 1 AA and 3 AA (Figure 5 I–L). Another interesting observation centered around two antiparallel β-strands, with β-strand E and F tolerating a 2 AA and 3 AA deletion, respectively (Figure 5 K, L). To understand the structural consequences of these deletions, we utilized the AlphaFold2 structural prediction framework ^35^. Predictions of VP1 wild-type structure showed similarities to the cryo-EM resolved EV-A71 virion, particularly at the surface-exposed loops (Figure 5 M). This enabled a comparison between the predicted VP1 structure and deletion mutants. The predicted mutant structures showed structural alterations, with the 2 AA deletion in β-strand E causing changes in the upstream DE loop. Conversely, the 3 AA deletion in strand F impacted the downstream FG loop (Figure 5 N, O). Subsequent Root Mean Square Deviation (RMSD) calculation comparisons between wild-type and deletion structures showed a deviation at these regions validating our observations (Figure 5 P, Q).

These structural analyses provided an interpretation of InDel fitness effects in the structural context of viral proteins. AlphaFold2 predictions for structural modifications caused by InDels will serve as a useful comparison for future investigations into the effect of InDels on viral proteins.

### InDels as a contributor to Enterovirus A species evolution

**Figure 6.**
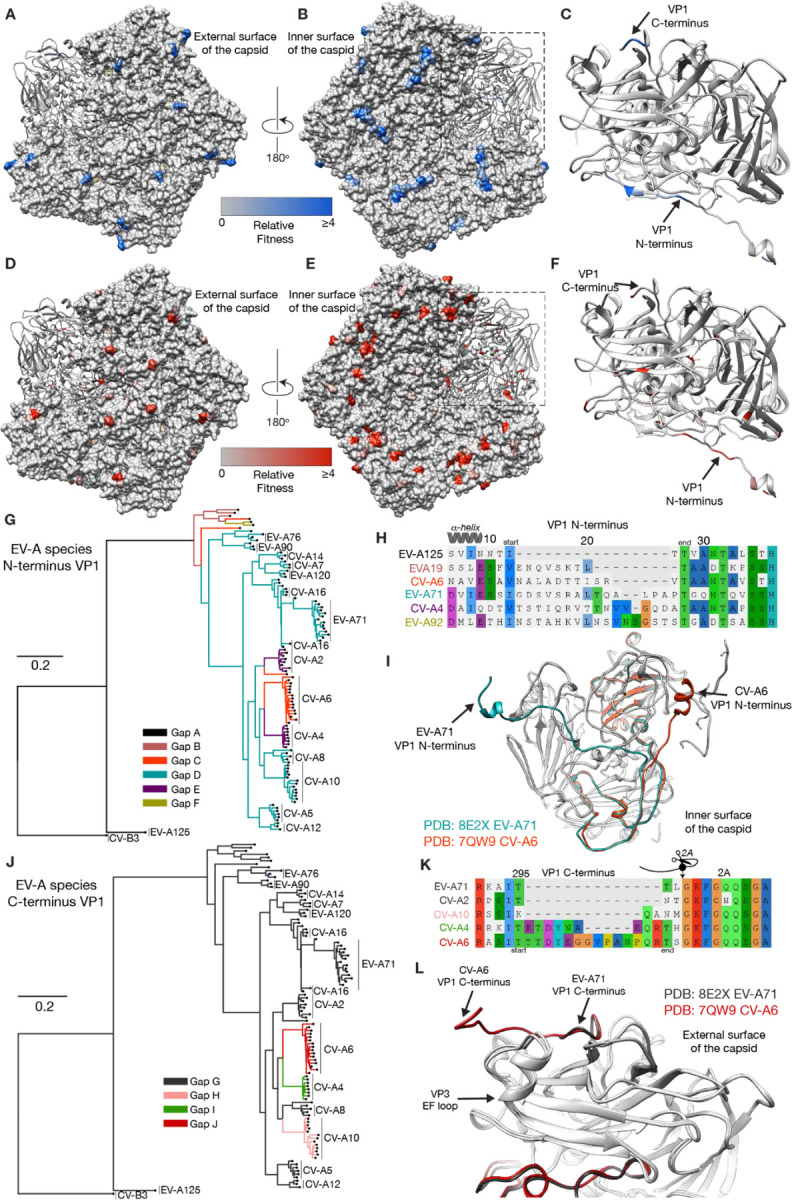
InDels as a contributor of Enterovirus A species evolution Pentamer of EV-A71 where insertions (a-c) or deletions (d-f) fitness values are mapped. Phylogenetic tree based on the full coding sequences of EV-A species (n= 107 sequences) was inferred using the maximum-likelihood method (RAxML software) with 100 bootstrap replicates. The phylogenetic tree is colored by the type of gaps observed at the N- (g) and C- (j) termini of VP1. Multiple sequence alignments of the N- (h) and C- (k) termini of VP1. Structural alignment of EV-A71 (PDB:8E2X) and CV-A6 (PDB:7QW9) pentamer at the N- (i) and C- termini (l).

We performed multiple sequence alignment using MAFFT of all complete EV-A proteomes (n= 107 sequences) to put our results in the context of the evolution of EV-A species. We compared the evolutionary potential of the EV-A proteome, as measured in our screen, to naturally occurring InDels in the EV-A species to characterize their contribution to the modern EV-A diversity. In this analysis, VP1 had the greatest number of gapped sites (eight) compared to all other capsid proteins (five). In our deep InDel scanning, the best tolerant sites in VP1 were present at the protein’s N- and C- terminus (Fig. 6 A–F). To understand the evolutionary history of these InDels, we generated a phylogenetic tree from our multiple sequence alignment using RAxML which employs a maximum-likelihood method for tree inference, with 100 bootstrap replicates (Fig. 6 G, J). Annotation of the phylogenetic tree by gap length allowed us to understand the emergence of InDels across EV-A species evolution.

This showed a complex evolutionary history at the N-terminus of VP1, with six different gap lengths detected (Fig. 6 G, H). EV-A125, basal to the tree, had the longest gap length A (length=16). On the distal branches, insertions have been accumulating, with the gap length D (length=2) being the most common. Interestingly, a switch from gap length D to shorter or longer gaps has occurred multiple times along the phylogenetic tree. This type of InDel dynamics was unique to this site of the EV-A proteome. CVA-6 and EV-A71 VP1 N-termini both appeared as linear epitopes at the inner surface of the capsid but varied in length (Fig. 6 I). The evolutionary history of InDels at the C-terminus of VP1 followed a simple pattern. Most EV-A species had a gap length G (length=14), including non-human Enteroviruses and most circulating human Enteroviruses (Fig. 6 J). An insertion event of different lengths occurred prior to the emergence of CV-A4 and CV-A6 (Fig. 6 J, K). This insertion in the CV-A6 VP1 C-terminus led to the extension of the C-terminal end and covered the EF-loop of VP3 (Fig. 6 L). These data highlight the unique roles of InDels in shaping the structural plasticity of VP1 throughout EV-A virus evolution.

## Discussion

InDels have shaped the evolution of RNA viruses by leading to the gain and loss of viral genes ^36^. InDels impact viral protein architecture and have directly contributed to the emergence of several pathogenic RNA viruses, such as SARS-CoV-2 and Rhinovirus C. However, the mutational landscape explored experimentally in RNA viruses has been limited to non-synonymous mutations. In this work, we expand the experimental toolkit in RNA viruses by adapting newly developed deep InDel scanning pipelines (SPINE and DIMPLE) to explore deletional and insertional tolerance at single-residue resolution. We generated the most comprehensive map of InDel fitness effects in a viral proteome, including over 45,000 insertions and 6,000 deletions. The distribution of mutational fitness effects revealed that >90% of InDels were lethal with several hotspot regions showing tolerance. Our calculated distribution of fitness effects aligns well with calculations derived from recent comprehensive deep sequencing of InDel diversity poliovirus-infected and dengue-infected cells ^2^.

The programmable nature of our adapted pipelines allowed the characterization of the detected hotspot in terms of amino acid specificity and deletion lengths. The changes in fitness effects when modulating InDel types could reflect context-specific factors affecting viral protein localization, stability, and enzymatic activity. This aspect of our work is unique compared to other attempts in performing deep InDel scanning in RNA viruses. Previous studies did not screen for deletions and only assessed the effects of one insertion type ^20,37^.

The detected hotspot regions in EV-A71 were primarily located in 2A(pro), the viral protease responsible for capsid maturation, and VP1, one of the major capsid proteins. Mapping the tolerance of InDels on the 2A(pro) revealed the core structural constraints acting on this viral enzyme. Tolerated InDels avoided the active site and zinc finger binding domain, which are both crucial for its activity ^32^. We also identified constraints reflecting host-virus interactions. For example, the binding interface with the host factor SETD3 did not tolerate InDels, confirming this interaction is crucial for viral replication ^38^.

These principles of constraint were also apparent from the analysis of the secondary structural elements in 2A(pro), which revealed that loops demonstrate greater tolerance to InDels than β-strands and helices. Most notably, the bII2-cII loop in 2A(pro) was highly tolerant to InDels, consistent with its highly polymorphic structure, which is both structurally dynamic and highly divergent across Enteroviruses. In CV-B4, bII2-cII loop tip is shifted downwards by around 7Å compared to EV-A71. In addition, the EV-A71 bII2-cII loop is proposed to undergo conformational changes upon substrate entry into the viral protease ^39^. The remarkable mutational plasticity of 2A(pro) may reflect its role as a soluble protease with fewer protein-protein interactions, rather than a membrane-associated component of the highly-coordinated replication complex. Notably, in our multiple sequence alignment analysis, we did not detect gaps in 2A(pro), suggesting that 2A(pro) may evolve under relaxed selection relative to the other regions of the EV-A71 proteome where InDels are more pervasive across EV-A.

Finally, we focused on the capsid proteins. Capsid proteins thread a difficult evolutionary needle. They maintain an extracellular, metastable state that encloses and protects the genome in harsh conditions within and between hosts, but that is labile enough to rapidly uncoat after engaging host receptors or host environments. Capsid proteins, specifically VP1, contain key immune epitopes that must evolve rapidly to evade immune pressures. VP1 had the highest number of beneficial variants. This corresponds well with our multiple sequence alignment, where VP1 had the highest number of gaps. In our InDel screen of VP1, the most tolerant regions were at the N- and C-termini. Phylogenetics analysis on the C-terminus of VP1 revealed relatively simple InDel dynamics where insertions occurred in CVA-4 and CVA-6. Since the cryo-EM structure of CV-A6 has been recently resolved, we could structurally compare it with the EV-A71 virion. We observed that this insertion extended, covering the VP3 EF loop. This has been proposed to change interactions with the immune system ^40^.

Despite significant structural and functional constraints acting upon the capsid, the N-terminus of VP1 has undergone multiple insertions and deletions along the evolutionary descent of EV-A. These historical InDel dynamics were unique to VP1, suggesting that strong selective pressures have driven frequent InDel events in this region. The sites most affected by these mutations are concealed within the mature capsid, however, counterintuitively, this region has also been described as highly immunogenic ^41–44^. This illustrates the critical host- and virus-facing functions of VP1, which place conflicting selective pressures on this region. The mature virion of Enteroviruses has been shown to undergo transient conformational changes termed “viral breathing”, where the major structural rearrangement is the reversible exposure of the VP1 N-terminus to the external surface ^45,46^. During the virus uncoating, pH and entry host factors trigger the irreversible externalization of the N-terminus of VP1 and VP4 release leading to the formation of the A particle, an intermediary state of virus entry ^47,48^. This information places the VP1 N-terminus at the center of Enterovirus entry and immune engagement. We speculate that the structural flexibility of this region is a phenotypic trait acquired by EV-A species, allowing it to overcome immune bottlenecks and re-emerge in populations ^4^. Further characterization of the interactions of InDels in this region with the innate and adaptive immune system should be investigated.

Information on this additional layer of mutational constraint is critical for efforts to understand immune recognition and evasion, and to engineer immune responses to Enteroviruses, with the ultimate goal of developing a pan-Enterovirus vaccine. At a basic level, pan-Enterovirus vaccine design should consider the differences in InDels and InDel tolerance between different Enterovirus capsids. Additionally, these data could inform vaccine engineering approaches, for example, switching of surface exposed loops in VP1 has been recently demonstrated as an innovative strategy to design vaccines that could be effective against EV-A71 and CV-A16 ^25^. Our InDel scanning strategy reveals regions that tolerate better loop switching, potentially informing such engineering approaches.

The mechanisms underlying small InDels, i.e. polymerase slippage ^49^, are distinct from those generating large InDels, such as domain insertions or large deletions, which are more likely due to recombination events, which are pervasive in Enteroviruses ^50^. Since our work utilized reverse genetics systems for the generation of InDels, we did not study the mechanisms of InDel formation. In the future, it will be important to connect this work on the biological constraints of mutation to the detailed understanding of mutational mechanisms in Enteroviruses to develop a complete understanding of mutational and selective forces acting on these pathogens.

## Materials and Methods

### Cells and Reagents

All infection experiments were performed in Rhabdomyosarcoma (RD) cells (ATCC,CCL-136) grown at 37°C with 5% CO_2_ cultured in Dulbecco’s Modified Eagle Medium (DMEM) (ATCC, 30-2002) supplemented with 5% Fetal Bovine Serum (FBS) (gibco, 10437-028).

### Generation of domesticated EV-A71 Tainan virus

Because SPINE and DIMPLE use BsaI and BsmBI type IIS restriction enzymes to assemble mutant libraries, we first ‘domesticated’ the original EV-A71 strain Tainan/4643/98 molecular clone (Genbank accession: AF304458.1) ^51^ by removing ten BsmBI or BsaI sites to improve efficiency of downstream assembly steps. Parts of the molecular clone which did not include BsaI and BsmBI restriction sites were generated by PCR. In parallel, fragments containing the restriction sites were ordered as gene fragments (Twist Biosciences). All the fragments (six in total) had a 30 bp overlap and were assembled by NEBuilder HiFi DNA Assembly (New England Biolabs, E5520S). We compared virus production between the original and domesticated clones and observed that viral growth in RD cells was comparable with rescued virus titers reaching around 10^6^ TCID_50_/mL.

### Generation of EV-A71 Tainan InDel libraries

#### SPINE and DIMPLE library design

The SPINE and DIMPLE pipelines ^21,22^ were used for the design of the EV-A71 InDel libraries. A FASTA file containing the sequence of the domesticated EV-A71 molecular clone was provided where the first and last codon of the structural (P1) and non-structural (P2-P3) proteins were specified. The following line for SPINE was used to run the code for insertional libraries: >python3 run_spine.py -wDir inputdirectory -geneFile bbfree.fasta -oligoLen 300 -mutationType DIS. For DIMPLE, we used the GUI interface to generate deletion libraries. The SPINE pipeline split the P1 and P2-P3 libraries to 11 and 17 sub-libraries respectively. DIMPLE split the P1 and P2-P3 libraries to 18 and 27 sub-libraries respectively. This generated i.) oligopools containing an insertional handle or deletions (1,2 and 3 amino acids) at each possible position in the modified regions, and ii.) primers for inverse PCR and oligopool amplification. Inward facing BsmBI sites are present in the primers and the oligopool allowing cloning of the oligopool into the plasmid backbone. The insertional handle contains two outward facing BsaI sites that will be used for subsequent insertion of sequences of interest.

#### Molecular biology pipeline

Inverse PCR fragments were amplified through the Q5 High-Fidelity DNA Polymerase (New England Biolabs, M0491L), DpnI (New England Biolabs, R0176L) digested for 1 hr at 37°C and then gel purified using Monarch DNA Gel Extraction Kit (New England Biolabs,T1020L). Oligopool amplification was performed using the KAPA HiFi HotStart PCR Kit (Roche, KK2502) and ran on the Agilent TapeStation 4200 using High Sensitivity D1000 ScreenTapes (Agilent, 5067-5584) to verify the presence of one single peak of the correct length. Then, 300 ng of the inverse PCR product was mixed with 20 ng of the corresponding oligopool in a NEBridge BsmBI-v2 Golden Gate assembly reaction (New England Biolabs, E1602L) and cycled through 60 cycles of digestion at 42 °C and ligation at 16 °C and finally incubated for 5 min at 65 °C to remove any unligated products. Ligation products were cleaned up using Monarch PCR & DNA Cleanup Kit (New England Biolabs, T1030L), eluted in 7 µl of water, and then 1 µl was transformed in NEB 10-beta Electrocompetent E. coli (High Efficiency) (New England Biolabs, C3019I) according to manufacturer conditions. Transformed cells were grown in a 50 mL LB media culture containing 100 µg/mL of carbenicillin (ThermoFisher Scientific, J67159.AE) at 37 °C for 14 hrs and then purified using the QIAGEN Plasmid Midiprep kit (QIAGEN, 12145). A small subset of the transformed cells were plated on carbenicillin plates to assess transformation efficiency and establish optimal coverage. The sub-libraries were mixed in equimolar fashion to generate either P1 or P2-P3 InDel libraries.

#### Generation of insertional libraries with different inserted residues

To remove any contaminating wild-type molecular clone, a Chloramphenicol cassette was designed flanked with i.) inward facing BsaI sequences to replace the insertional handle, and ii.) outward facing BsmBI recognition sequences to replace the antibiotic resistance cassette with a tag of interest. To accomplish that, 300 ng of the structural P1 or non-structural P2-P3 insertional handle library was mixed with 20 ng of the Chloramphenicol cassette in a NEBridge BsaI-v2 Golden Gate assembly reaction (New England Biolabs, E1601L) and cycled through 30 cycles of digestion at 37 °C and ligation at 16 °C and finally incubated for 5 min at 65 °C to get rid of any unligated products. Ligation products were cleaned up using Monarch PCR & DNA Cleanup Kit, eluted in 7 µl of water, and then 1 µl was transformed in NEB 10-beta Electrocompetent *E. coli* according to manufacturer conditions. Transformed cells were grown in a 50 mL LB media culture containing 100 µg/mL of carbenicillin and 25 µg/mL of Chloramphenicol at 37 °C for 14 hrs and then purified using the QIAGEN Plasmid Midiprep kit. A small subset of the transformed cells were plated to assess transformation efficiency and establish optimal coverage. To replace the antibiotic cassette with an insertion of interest, 300 ng of the P1 or P2-P3 Chloramphenicol insertional library was mixed with 20 ng of BsmBI-flanked DNA fragments in a BsmBI assembly reaction and followed the previously described protocol for purification of the plasmid libraries.

#### Virus rescue

For virus rescue (passage 0 virus), the EV-A71 molecular clone was linearized downstream of the poly(A) tail using the enzyme EagI-HF (New England Biolabs, R3505L) at 37 °C overnight. Linearized plasmid was cleaned up using the Monarch PCR & DNA Cleanup Kit and used as a template for *in-vitro* transcription with the HiScribe T7 High Yield RNA Synthesis Kit (New England Biolabs, E2040S). The *in-vitro* transcription product was treated with DNase I (New England Biolab, M0303L) for 10 minutes at 37°C and cleaned up using the Monarch RNA Cleanup Kit (New England Biolabs, T2040L). Then, viral RNA was transfected into RD cells using the TransIT-mRNA Transfection Kit (mirus bio, MIR 2250) using 0.5x of the recommended RNA and reagent concentrations. After two days of transfection, cells were subjected to two freeze-thaw cycles. To remove cellular debris, the supernatant was centrifuged at 2,000 x g for 5 minutes. Virus rescue efficiency was evaluated by titrating the supernatant using TCID50 ^52^. EV-A71 molecular clone produces ~10^6^ TCID_50_/mL. InDel libraries virus rescue efficiency varied between 10^3^–10^4^ TCID_50_/mL for insertion libraries and 10^5^–10^6^ TCID_50_/mL for deletion libraries.

#### Virus passaging and sequencing

For passage of rescued InDel libraries, RD cells were washed once with PBS (ATCC, 30-2200) and then incubated with low inoculum (MOI≤0.1) of rescued virus for 1 hr at 37 °C. Then, media was supplemented to the cells and infection was allowed to continue either for 24 hrs for the generation of a new passage or 9 hrs for intracellular RNA extraction and subsequent sequencing. Intracellular RNA extraction was performed using the QIAGEN RNeasy kit (QIAGEN, 74106).

#### Sequencing pipeline

1 μg of sample RNA was used in a ProtoScript II reverse transcription reaction (New England Biolabs, E6560L) to produce cDNA by spiking in a gene-specific primer at the start of the 2A gene or in the 3’ UTR to allow the amplification of the P1 and P2-P3 regions of the genome respectively. Then, the cDNA or plasmid DNA was amplified to generate amplions using the Q5 High-Fidelity DNA Polymerase in four independent PCR reactions for 25 cycles to minimize PCR-derived errors. The amplicons were gel-purified using the Monarch DNA Gel Extraction Kit and used as inputs for Nanopore and Illumina sequencing library preparation. Nanopore sequencing libraries were prepared using the Native Barcoding Kit 24 V12 or V14 (Oxford Nanopore Technologies, SQK-NBD112.24 or SQK-NBD114.24) according to the manufacturer’s instructions and were run on a MinION device. Illumina sequencing libraries were prepared using the Twist Biosciences Enzymatic Fragmentation 2.0 kit with universal adapters (Twist Biosciences, 104207) targeting fragment sizes in the range 180–220 bp. Illumina libraries were run on a MiSeq device using the MiSeq reagent kit v2, 300-cycles (Illumina, MS-102-2002).

### Computational pipeline

#### Mapping insertions and deletions

Nanopore sequencing reads were basecalled using the high-accuracy module of the neural network basecaller guppy (guppy_gpu/6.0.6), producing fastq files from fast5 files for each sample. For Illumina sequencing reads, bcl2fastq2 was run, transforming bcl files to demultiplexed fastq files. All sequencing reads were mapped using minimap2 to generate sam files. Mapped sequencing reads were fed into two different computational pipelines called stickleback and smelt that map insertions and deletions respectively. All read data will be made available at under NCBI project number, PRJNA1066851

#### Structural analysis

The PDB structures used for the structural mapping were: PDB: 8POA (EV-A71 2A), PDB: 8E2X (EV-A71 virion), and PDB: 7QW9 (CV-A6 virion). Secondary structures assignment was performed using STRIDE’s web server ^33^. For mapping of InDel relative fitness values onto the structures, an “attribute” text file compatible with Chimera (version 1.16) was generated modifying the InDel positions to align with the structural information of the PDB file. Then, we used the “define attributes” function to apply the “attribute” text file to a given structure and “rendered by attribute”, using color to display relative fitness values. ColabFold v1.5.3 ^35^ using the AlphaFold2 module was used for the prediction of the EV-A71 VP1 structure and deletion variants. Biopython and numpy were used to import the PDB coordinates of predicted VP1 structures and calculate the RMSD between the different variants.

#### Phylogenetics analysis

All complete Enterovirus A protein sequences were downloaded from NCBI virus, then clustered at 98% by cd-hit yielding 107 sequence clusters. The representative sequences derived from this clustering were then aligned by MAFFT. Next, the aligned sequences were indexed to the EV-A71 strain Tainan/4643/98 and the starting position of all gaps was recorded. These gap positions were then graphically mapped onto the experimental InDel screen results in R. A second MAFFT alignment was performed using the same 107 Enterovirus A clusters and the ICTV Enterovirus B exemplar isolate sequence (GenBank: AAB59927.1) as an outgroup. A phylogenetic tree was produced from this alignment using the maximum-likelihood method in RAxML with 100 bootstrap replications. The PROTGAMMAWAG option was selected in RAxML which is for amino acids with a gamma rate heterogeneity model using a WAG substitution matrix. FigTree was used for phylogenetic tree visualization and customization. AliView was used for visualization and representation of the multiple sequence alignment.

#### Statistics, reproducibility, and data analysis

Statistical data analysis and visualization was performed with R and Graphpad Prism 9. Base R code was used for Chi-squared analysis. For Lorenz curves and calculation of Gini coefficients, the R library ‘ineq’ was used. Analysis scripts and supplementary dataframes are all deposited in the Dryad repository (https://doi.org/10.5061/dryad.866t1g1xm). Insertion and deletion experiments in Figure 2 are means of three biological replicates. Experiments regarding the insertion libraries with different inserted residues in Figure 3 are means of two biological replicates.

## Supplementary Material

Supplement 1
