# Supplementary material for: Uncovering Structural Plasticity of Enterovirus A through Deep Insertional and Deletional Scanning
Source: Res Sq. 2024 Jan 24:rs.3.rs-3835307. Preprint. [Version 1] doi: 10.21203/rs.3.rs-3835307/v1 (PMC10896406; doi:10.21203/rs.3.rs-3835307/v1)
Supplement: Supplement 1 [file NIHPPrs3835307v1-supplement-1.pdf]

Supplementary Figures

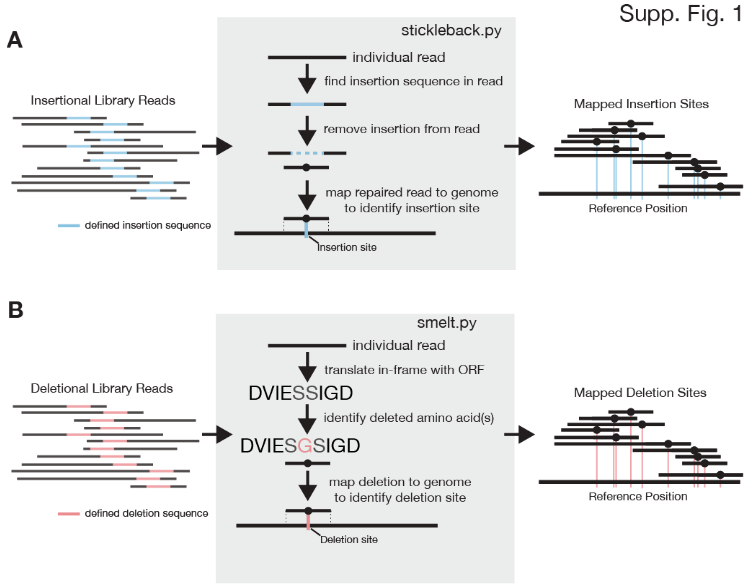

Supplementary Figure 1. Computational pipeline for mapping of InDels *(a) Stickleback searches for a user-specified insert sequence from sequencing reads and then removes the insertion from the read and maps it back to the viral genome identifying the insertion site. The output is a .csv file with mapped insertion sites to the reference genome. (b) Smelt translates all sequencing reads in-frame of the open reading frame of the viral genome. Then, smelt will identify deletion of amino acid(s) and map the deletion site back to the reference genome. The output is a .csv file with mapped deletion sites to the reference genome. Users can specify the size of the deletion to be detected.*

Supp. Fig. 2

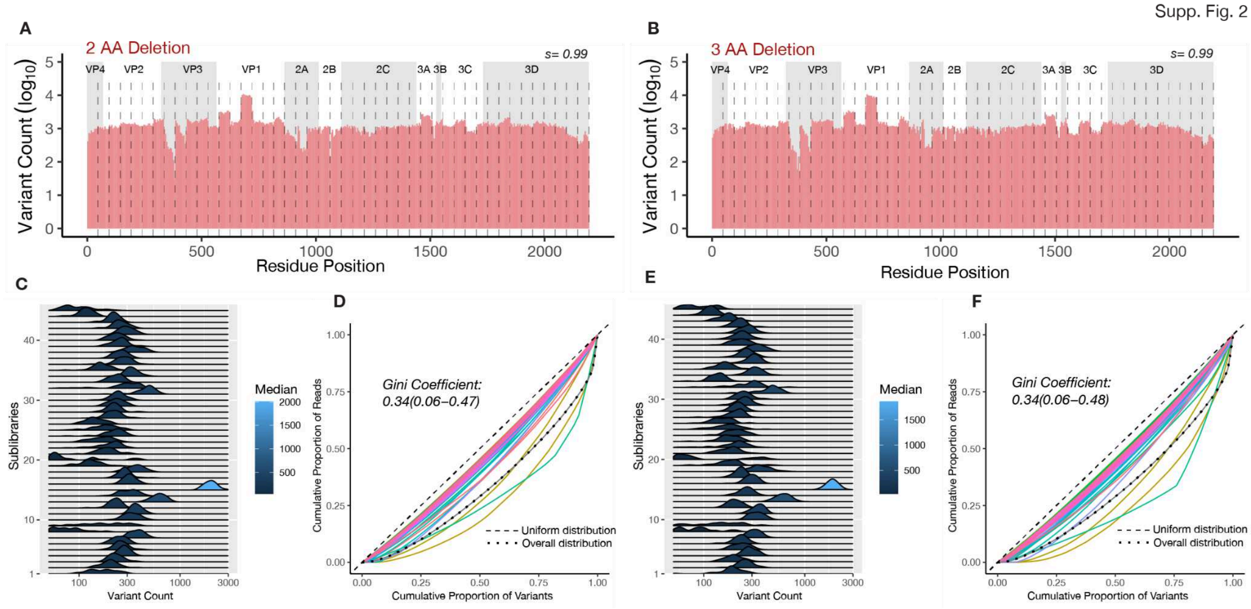

Supplementary Figure 2. Deep Deletion scanning of the EV-A71 proteome *Barplot (bin=5) showing the variant count across the viral proteome for 2 AA (a) and 3 AA (b) deletion. The distribution of variants is shown through ridge kernel line plots and Lorenz curves for 2 AA deletions (c,d) and 3 AA deletions (e,f).*

Supp. Fig. 3

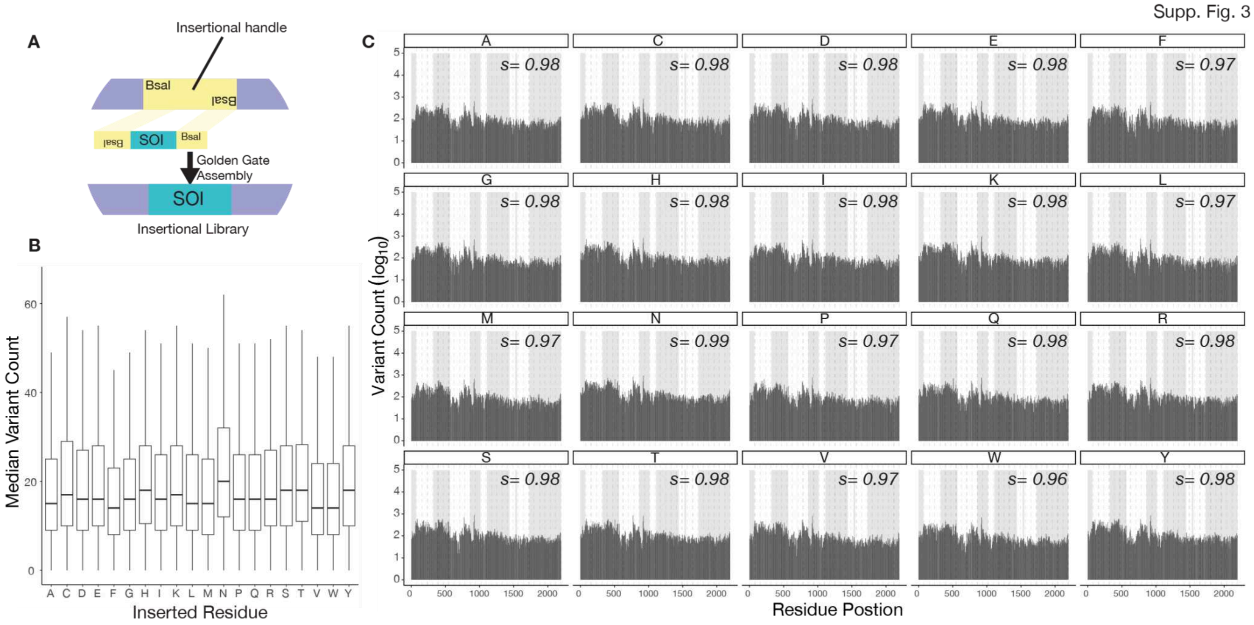

Supplementary Figure 3. Deep Insertion scanning of the EV-A71 proteome *(a) Schematic of the insertional handle that is useful for the generation of new insertional libraries with any sequence of*

interest. (b) Boxplots of the median variant count for the insert residues. (c) Barplots (bin=5) of the variant count for all inserted residues across the viral proteome.

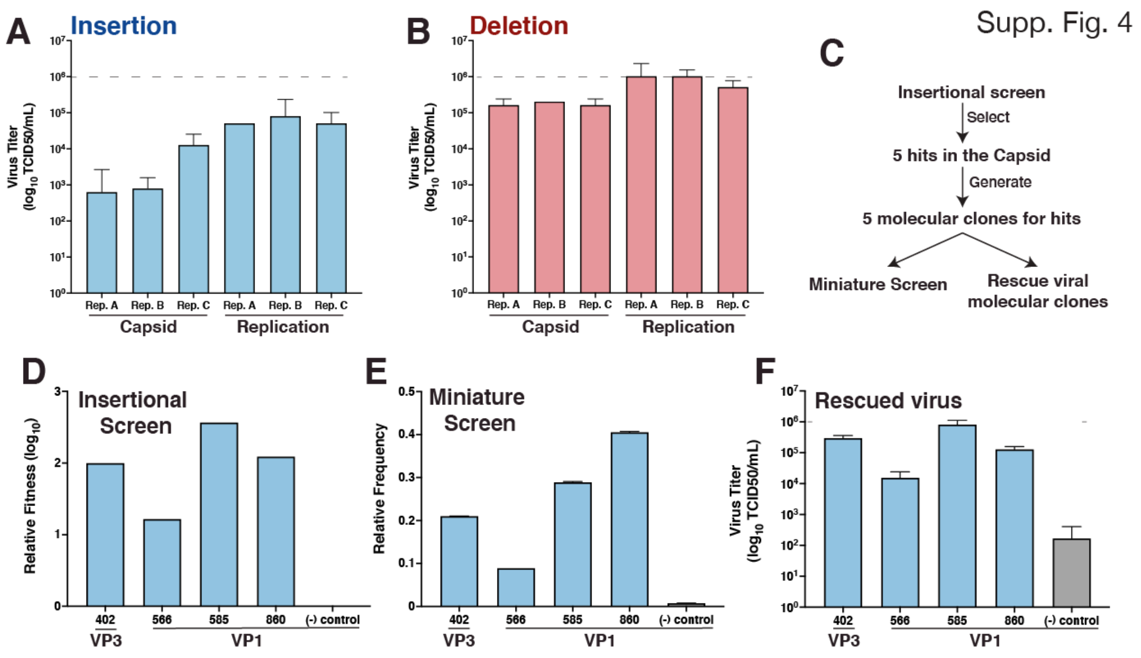

Supplementary Figure 4. Validation of insertional handle dataset *Passage 0 virus rescue efficiency is shown through TCID50 for insertion (a) and deletion libraries (b). (c) Schematic for the pipeline of selection for insertions to validate through either a miniature screen or by rescuing individual molecular clones. (d) Bar graph showing the relative fitness values from the insertional screen for the five selected insertions. (e) Relative frequency value calculations derived from a miniature screen with the five insertions is shown in this bar graph. (f) Passage 0 virus rescue efficiency for the five insertions at different positions of the capsid proteins.*
